# A20 Haploinsufficiency in East Asia

**DOI:** 10.3389/fimmu.2021.780689

**Published:** 2021-11-26

**Authors:** Tomonori Kadowaki, Saori Kadowaki, Hidenori Ohnishi

**Affiliations:** ^1^ Department of Infection and Immunity, Aichi Children’s Health and Medical Center, Aichi, Japan; ^2^ Department of Pediatrics, Graduate School of Medicine, Gifu University, Gifu, Japan; ^3^ Clinical Genetics Center, Gifu University Hospital, Gifu, Japan

**Keywords:** autoimmune disease, autoinflammatory disease, A20 haploinsufficiency, East Asia, *TNFAIP3*

## Abstract

A20, encoded by the *TNFAIP3* gene, is a negative regulator of tumor necrosis factor (TNF)-nuclear factor-κB signaling. It was recently demonstrated that A20 haploinsufficiency (HA20), caused by a heterozygous mutation in the *TNFAIP3* gene, can present as an early onset autoinflammatory disease resembling Behçet’s disease (BD). In addition to autoinflammatory symptoms, HA20 was also reported to be associated with autoimmune diseases and immunodeficiency. Because the phenotypes associated with HA20 are broad, with different severities observed even among individuals in the same family with identical mutations, it has been assumed that the symptoms of HA20 may depend on genetic background and environmental factors. In this review, we summarize the characteristics of patients with HA20 in East Asia and compare these with patients in other regions, mainly the USA and Europe. Patients with HA20 in East Asia developed recurrent fever more frequently than patients in other regions, but were less likely to develop typical BD symptoms such as skin rashes and genital ulcers. In addition, patients with HA20 in East Asia had low rates of complication with autoimmune diseases and low autoantibody detection rates. While anti-TNF-α agents were the primary treatments for severe HA20 in East Asia, anti-interleukin-1 agents and Janus kinase inhibitors were also administered in other regions. Future studies will need to establish methods for analyzing the pathophysiology of HA20 and determining optimal treatment strategies for each patient.

## Introduction

Behçet’s disease (BD) is a chronic inflammatory disease first described by Hulusi Behçet in 1937. BD is characterized by recurrent aphthous stomatitis, skin lesions, uveitis, and genital ulcers (1). Gastrointestinal, cardiovascular, and central nervous system symptoms can also occur in patients with BD. BD is also called the Silk Road disease and has higher prevalence in the Mediterranean, the Middle East, and East Asia compared with other regions. BD shows familial clustering, and thus it has been proposed that genetic predisposition might be a major factor in pathogenesis. Associations between BD and human leukocyte antigen (HLA) genes and polymorphisms, especially HLA-B51, are well established. Recent genome-wide association studies (GWAS) reported that polymorphisms in *IL-10*, *IL-23R*, and *IL-12RB2* were associated with risk of developing BD; however, none contributed directly to the onset of BD (2) with high penetrance. In 2016, A20 haploinsufficiency (HA20), caused by heterozygous mutation of the *TNFAIP3* gene, was reported to cause an early-onset autoinflammatory disease presenting with BD-like features such as recurrent aphthous stomatitis, genital ulcers, and gastrointestinal symptoms (3). Since that time, numerous cases with varied clinical presentation have been reported all over the world, especially in East Asia including China and Japan. Several studies have investigated the function of A20, the pathophysiology of HA20, and optimal treatment strategies. Furthermore, patients with HA20 can present with both autoinflammatory BD-like symptoms as well as autoimmune symptoms and/or immunodeficiency. Variation in symptoms, disease severity, and response to treatment occurs even in individuals within a single family carrying identical mutations. To describe the characteristics of HA20 in East Asia and future prospects for treatment of HA20, we reviewed the published literature on HA20 in East Asia and other regions, focusing on disease manifestations, complications, treatments, and distribution of *TNFAIP3* mutations.

## Function of A20

A20 is encoded by the *TNFAIP3* gene located on chromosome 6q23.3. A20 is a negative regulator of tumor necrosis factor (TNF)-nuclear factor (NF)-κB signaling. In the NF-κB signaling pathway, signals transmitted from TNF-α, interleukin (IL)-1 family members, Toll-like receptors (TLRs), B-cell receptors (BCRs), and T-cell receptors result in ubiquitination of factors in each pathway, NF-κB activation, and production of inflammatory cytokines (4). A20 has an ovarian tumor (OTU) domain in the N-terminal region and seven zinc finger (ZnF) domains in the C-terminal region. The OTU domain deubiquitinates K63 polyubiquitin chains from receptor interacting protein 1, TNF receptor-associated factor (TRAF) 6, and IκB kinase (IKK) γ (5–7) resulting in suppression of signaling. In addition, the ZnF4 domain has E3 ligase function and supports polyubiquitination with K48 polyubiquitin chains, inducing degradation by the proteasome (6). The ZnF7 domain binds the TNF receptor complex *via* linear polyubiquitin chains and inhibits the formation of the linear ubiquitin chain assembly complex and the IKK complex (8). Post­translational modifications of A20 have suppressive effects on this pathway: for instance, IKKβ-mediated serine phosphorylation near the ZnF domains (notably at Ser381) promotes A20-mediated cleavage of K63-linked polyubiquitin chains and enhances the inhibitory activity of A20 (9). These functions of A20 contribute to inhibition of NF-κB signaling from each receptor.

A20 also functions to regulate the interferon regulatory factor (IRF) pathway following pathogen recognition. In this pathway, the pattern recognition receptors retinoic acid-inducible gene I and melanoma differentiation-associated protein 5 recognize viral nucleic acids and activate mitochondrial antiviral signaling protein and TRAF3. Subsequently, they combine with TANK-binding kinase 1 (TBK1) and IKKi and are activated by ubiquitination with K63 polyubiquitin chains. Activation of these kinases results in phosphorylation, dimerization, and nuclear translocation of the transcription factor IRF3, followed by transcriptional activation of the type 1 interferon (IFN) gene. Type 1 IFN binds to the IFN-α/β receptor, activates Janus kinase (JAK)-signal transducer and activator of transcription signaling, and induces transcription of IFN-inducible-genes (ISGs). A20 inhibits the IRF pathway and IFN responses by cleaving K63 polyubiquitin chains from TBK1/IKKi (4). In patients with HA20, disrupted suppression of signal transduction results in increased production of inflammatory cytokines and type 1 IFNs followed by autoinflammatory symptoms.

## Patients With HA20 in East Asia and Other Regions

We summarized the literature on patients with HA20 resulting from *TNFAIP3* mutations starting from the first report of this disease in 2016 until August 2021. A total of 20 studies in East Asia and 15 studies in other regions, mainly the USA and Europe, were published during this period (10–44). Case reports from East Asia were analyzed separately from those in other regions. We focused on major BD-like symptoms, development of autoimmune diseases, non-autoimmune complications, and treatments administered. The following East Asian patients with HA20 were excluded: one patient’s gender was not stated, three patients did not have detailed symptom information, and two of these three patients were not investigated for autoimmune diseases and/or autoantibodies. A single patient described by multiple studies was counted as one case. The prevalence of HA20 symptoms was compared between East Asia and other regions using Fisher’s exact test in Prism 7 (GraphPad Software, San Diego, CA, USA). Values of *p* < 0.05 were considered statistically significant.

**Table 1** shows the symptoms of HA20 patients in East Asia and other regions. A total of 74 patients in 39 families were affected by HA20 in East Asia, while a total of 51 patients in 23 families were affected by HA20 in other regions. The age of onset ranged from neonatal to around 30 years in both groups. Thus, the age of onset of HA20 appears to be younger than that of BD.

**Table 1:** The comparison of symptoms and treatment between East Asia versus other countries.

	Countries		*p*-value
	East Asia	without East Asia	
Counts of family	39	23	
Counts of Patients	74	51	
Number of male patients: n/total patients (%)	35/73 (47.9%)	15/51 (29.4%)	
Range of onset age	neonatal period to 32 y	2 mo to 29 y	
**Symptoms: n/total patients (%)**			
Recurrent stomatitis	57/71 (80.2%)	42/51 (82.6%)	0.819
Cutaneous lesions	23/71 (32.4%)	27/51 (52.9%)	0.026
Ocular symptoms	3/71 (4.2%)	5/51 (9.8%)	0.277
Genital ulcers	27/71 (38.0%)	34/51 (66.7%)	0.003
Arthritis	23/71 (32.4%)	22/51 (43.1%)	0.257
Abdominal symptoms	40/71 (56.3%)	28/51 (54.9%)	>1.000
Cardiovascular lesion	2/71 (2.8%)	5/51 (9.8%)	0.128
Central nervous system symptoms	3/71 (4.2%)	2/51 (3.9%)	>1.000
Pathergy	2/71 (2.8%)	4/51 (7.8%)	0.235
Recurrent fever	50/71 (70.4%)	19/51 (37.3%)	<0.001
Criteria of ISGFBD 1990			
fulfil/total patients (%)	18/71 (25.4%)	19/51 (37.3%)	0.169
Number of autoimmune diseases and/or autoantibodies	21/72 (29.2%)	30/51 (58.8%)	0.002
Development of autoimmune diseases	Autoimmune thyroid disease, AIH, SLE, ALPS-U, PsA, Detection of autoantibodies	Autoimmune thyroid disease, SLE, ITP, type 1 DM, PsA, Detection of autoantibodies	
Symptoms that may be associated with HA20 without autoimmune diseases	IgA vasculitis, CH, IP, Lymphadenitis, Nephrotic syndrome, Aseptic meningitis, DD, MAS, HL, Craniopharyngioma, BCG dermatitis, Chronic active EBV infection	IgA vasculitis, CH, IP, Lymphadenitis, Cerebral infraction, Pancytopenia, IgG2 and IgG4 deficiency, Recurrent infection, Chronic active EBV infection, KD like Coronary vasculitis	
**Treatment: Number of used patients (%)**			
Colchicine	18 (24.3%)	18 (35.3%)	
Steroid	34 (45.9%)	19 (37.3%)	
Anti-TNF-α agents	21 (28.4%)	14 (27.5%)	
(infliximab, adalimumab, etanercept)			
Anti-IL-1 agents	1 (1.4%)	10 (19.6%)	
(anakinra, canakinumab, rilonacept)			
Tocilizumab	3 (4.1%)	3 (5.9%)	
Rituximab	1 (1.4%)	2 (3.9%)	
JAK inhibitor agents	1 (1.4%)	6 (11.8%)	
(tofacitinib, baricitinib)			
Hematopoietic cell transplantation	1 (1.4%)	2 (3.9%)	
Sirolimus	0 (0.0%)	1 (2.0%)	
NSAIDs	4 (5.4%)	1 (2.0%)	
Methotrexate	12 (16.2%)	7 (13.7%)	
Cyclophosphamide	3 (4.1%)	1 (2.0%)	
Cyclosporine A	6 (8.1%)	3 (5.9%)	
Tacrolimus	2 (2.7%)	1 (2.0%)	
Mycophenolate mofetil	4 (5.4%)	4 (7.8%)	
Azathioprine	3 (4.1%)	9 (17.6%)	
Mesalazine	12 (16.2%)	2 (3.9%)	
Thalidomide	8 (10.8%)	3 (5.9%)	
Hydroxychloroquine	3 (4.1%)	3 (5.9%)	
Dapsone	0 (0.0%)	2 (3.9%)	
Cimetidine	2 (2.7%)	0 (0.0%)	
Iguratimod	1 (1.4%)	0 (0.0%)	
Mizoribine	1 (1.4%)	0 (0.0%)	
IVIG	1 (1.4%)	2 (3.9%)	

AIH, Autoimmune hepatitis; ALPS-U, autoimmune lymphoproliferative syndrome undefined; BCG, Bacille de Calmette et Guérin; CH, chronic hepatis; DD, developmental disability; DM, diabetes mellitus; EBV, Epstein-Barr Virus; HL, Hodgkin lymphoma; Ig, immunoglobulin; IL, interleukin; IP, interstitial pneumonia; ISGFBD, International Study Group for Behçet’s disease; ITP, idiopathic thrombocytopenic purpura; IVIG, intravenous immunoglobulin; JAK, Janus kinase; KD, Kawasaki disease; MAS, Macrophage activation syndrome; NSAID, nonsteroidal anti-inflammatory drug; PsA, psoriatic arthritis; SLE, systemic lupus erythematosus; TNF, tumor necrosis factor.

## Locations of *TNFAIP3* Mutations in East Asia

**Figure 1:**
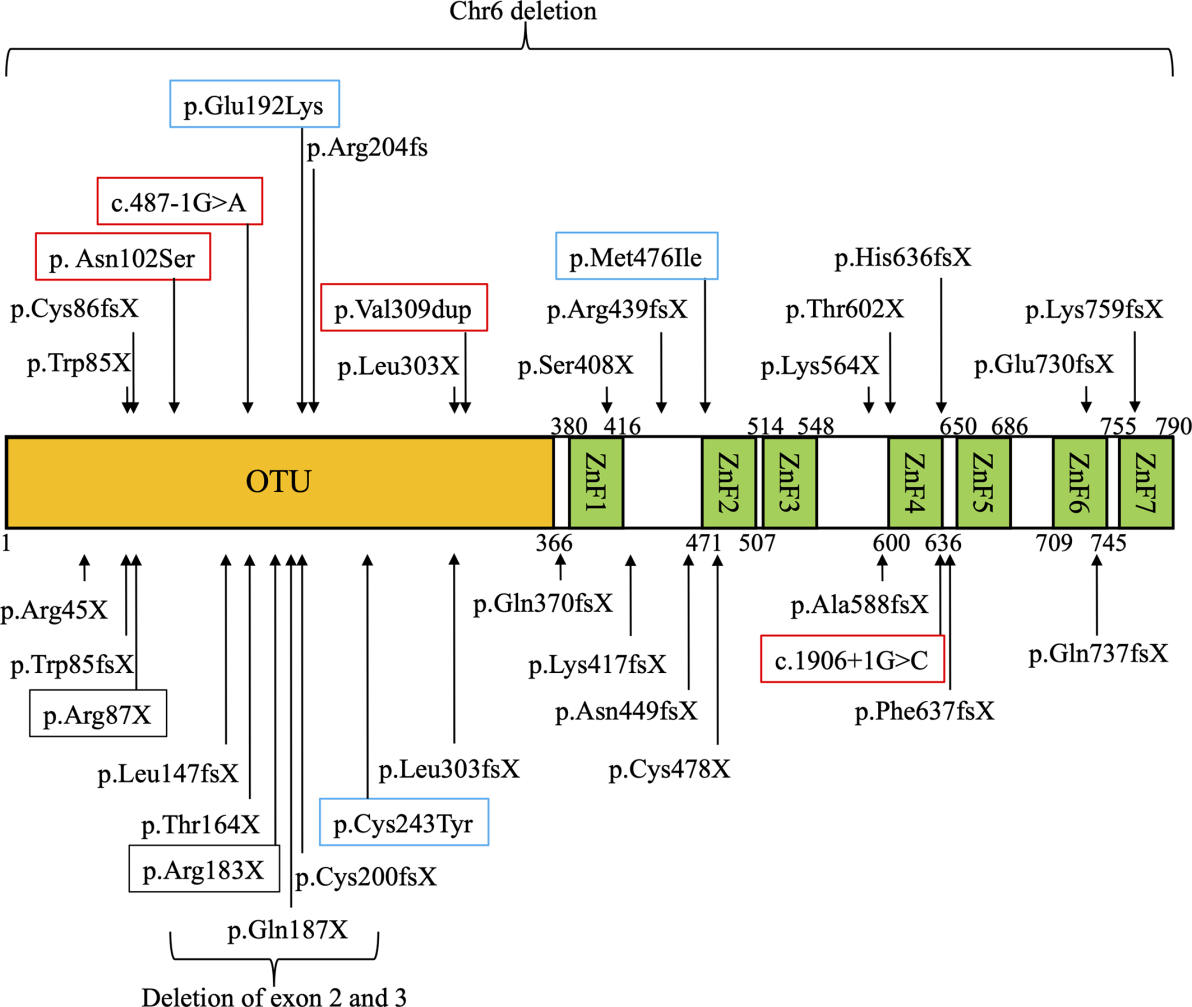
Domain structure of A20 and locations of *TNFAIP3* gene mutations in East Asian patients with A20 haploinsufficiency (HA20). Mutations of *TNFAIP3* reported in East Asia are indicated with arrows on the domain structure of A20. Three mutations whose pathogenic functions were evaluated by *in vitro* functional analysis are shown in blue squares. Four mutations whose pathogenic functions were not evaluated by *in vitro* functional analysis are shown in red squares. The mutations overlapping between different families are shown in black squares.

The domain structure of A20 and sites of mutations in *TNFAIP3* among East Asian patients with HA20 are shown in **Figure 1**. A20 mutations in patients with HA20 in East Asia were widely distributed from the OTU to the ZnF7 domains. Most mutations were truncating and included frameshift mutations, nonsense mutations, splice site mutations, and large deletions. However, some missense mutations were also reported. Three missense mutations that were judged as pathogenic *via* functional analyses are shown in blue squares. *TNFAIP3* Cys243Tyr (45) and Glu192Lys (18) were reported in Japan, while Met476Ile (20) was reported in China. Among previously reported mutations, the pathogenic significance of four potential splice site mutations or duplication mutations (shown in red squares) has not been evaluated using functional analyses. One of these mutations was reported in Japan (16), while four were reported in China (25, 27). Among the mutations in East Asia, Arg183X was found in two families (22, 27). The only mutation overlapping between East Asia and other regions was Arg87X, which was found in one family in each case (21, 42).

## Clinical Symptoms of HA20

**Table 1** shows the symptoms of patients with HA20 in East Asia and other regions. Recurrent stomatitis was the most common symptom in both groups of patients with a prevalence of 80.2% and 82.6%, respectively. In East Asia, the next most common symptoms of HA20 were recurrent fever, abdominal symptoms, genital ulcers, and skin rashes. In other regions, these symptoms were also common, albeit with different frequencies. Recurrent fever was significantly more common among East Asian patients compared with those in other regions (70.4% *vs.* 37.3%, *p* < 0.001). Interestingly, the major BD-like symptoms of genital ulcers and skin rash, including erythema nodules, folliculitis-like eruption, and thrombophlebitis, were more common among patients with HA20 in other regions compared with East Asia. There was no significant difference in the proportion of HA20 patients in East Asia and other regions who fulfilled the International Study Group for Behçet’s disease (ISGFBD) 1990 criteria (46). However, there was a trend toward higher frequency of meeting these criteria in other regions compared with East Asia (25.4% *vs.* 37.3%, *p* = 0.169).

## Complications Associated With HA20

HA20 has been reported to often lead to autoimmune diseases such as systemic lupus erythematosus (SLE) and autoimmune thyroid disease. We compared the autoimmune disease development rate and/or autoantibody positive rate in East Asia and other regions and found that these rates were significantly lower in East Asia (**Table 1**). Non-autoimmune complications were diverse and included IgA vasculitis, chronic hepatitis, and nephrotic syndrome. One patient with HA20 in East Asia developed Hodgkin lymphoma (10). Interestingly, persistent Epstein-Barr virus (EBV) infection occurred in patients with HA20 in both East Asia and other regions (20, 40). Furthermore, some patients in other regions developed immunodeficiency symptoms such as IgG2 and IgG4 deficiency, pancytopenia, and recurrent infection (30, 40, 41).

## Treatment of HA20

The number of patients with HA20 treated with medications and administration rates in East Asia and other regions are shown in **Table 1**. In both groups of patients, colchicine, systemic corticosteroids, disease-modifying drugs, and molecular targeted therapies were relatively commonly administered. In East Asia, anti-TNF-α agents (infliximab, adalimumab, and etanercept) were administered in most severe cases, while anti-IL-6 agents (tocilizumab) were also administered in some cases (10, 13, 16, 21, 24). Only one patient was treated with anti-IL-1 agents (canakinumab) in East Asia (47). In other regions, patients with severe HA20 who did not respond to anti-TNF-α agents were commonly treated with anti-IL-1 agents (anakinra, canakinumab, and rilonacept), anti-IL-6 agents, and JAK inhibitors (tofacitinib and baricitinib) (30, 32, 35, 36, 38–40). Rituximab was administered in a few patients with nephrotic syndrome or autoimmune diseases including SLE in East Asia and other regions (10, 31, 41). In addition, allogeneic hematopoietic cell transplantation (HCT) was also performed for one refractory patient in East Asia and one refractory patient in other regions (31, 47), and autologous HCT was performed for one patient in other regions (30).

## Discussion

This review summarizes the current status of HA20, including regional differences in clinical features, evaluation of the pathogenic significance of *TNFAIP3* variants, and treatment strategies.

We found that the proportions of patients with typical BD symptoms, such as skin rash and genital ulcers, and of patients who met the ISGFBD diagnostic criteria for BD, tended to be lower in East Asia compared with other regions. Additionally, the proportions of patients who were autoantibody-positive and/or who were developed autoimmune diseases were also significantly lower in East Asia compared with other regions. A multicenter study in Japan demonstrated that increased frequencies of double-negative T cells and follicular T cells contributed to the development of autoimmune diseases in patients with HA20 (48). Differences in autoimmune disease development rates between regions might be related to immunological differences associated with racial background. In addition, activation of type 1 IFN was reported to cause autoimmune diseases (49). HA20 patients in other regions were reported to show overexpression of ISGs (36, 38, 40, 43), while only one patient in East Asia showed elevated ISG expression (47). In this study, it is unclear whether the overexpression of ISGs is associated with regional differences in autoimmune disease because few patients were investigated for ISG expression. Future work should investigate the relationship between the expression of ISGs in HA20 and the development of autoimmune diseases. There are insufficient data to account for regional differences in the clinical features of HA20. Variation in the clinical features of HA20 were not completely explained by differences in mutation sites. It has been reported that musculoskeletal disorders were significantly more frequent among patients with mutations disrupting the ZnF domain of A20, whereas other symptoms were not affected by mutation site (50). Because genotype-phenotype correlations have not been demonstrated in HA20, variation in clinical features might be affected by other modifier genes and/or environmental factors. Some HA20 patients present with immunodeficiency symptoms such as hypogammaglobulinemia and persistent EBV infection (20, 30, 40). It was reported that mice with selective loss of A20 in B cells had fewer memory B cell and diminished levels of IgG1 and IgG3 (51). Moreover, in HA20 patients, it was suggested that over-activation of NF-κB signaling may cause T cell exhaustion and senescence and thereby decrease naïve Th cell frequency and facilitate persistent EBV infection (48). The composition of lymphocyte subsets may also be affected by age. We were unable to analyze age of onset in patients with HA20 because age of onset in some patients were not stated. Future studies will need to conduct detailed analyses of lymphocyte subsets in patients with HA20 and immunodeficiency to clarify the underlying pathophysiology.

Most HA20 mutations are truncating and include nonsense, frameshift, and splice site mutations. Three missense mutations have been reported in East Asia: *TNFAIP3* Glu192Lys, Cys243Tyr, and Met476Ile. While these missense mutations have demonstrated pathogenicity in *in vitro* functional analyses (18, 52), the pathogenic significance of other variants, such as p.Asn102Ser, c.487-1G>A, and p.Val309dup, has yet to be demonstrated *via* functional analyses. In particular, the frequency of the Asn102Ser allele is 0.01394 in East Asian according to the Genome Aggregation Database (gnomAD) dataset v.2.1.1 (https://gnomad.broadinstitute.org). Because HA20 is an autosomal dominant disease with high penetrance, *in vitro* functional analysis is essential to demonstrate that this variant with high allele frequency represents a pathogenic mutation. The analysis of NF-κB reporter gene activity has been commonly used as *in vitro* functional analysis to demonstrate the pathological significance of *TNFAIP3* variants, especially for truncating mutations (3, 10). Furthermore, it was reported that disruption of the inhibitory effects of *TNFAIP3* missense variants could be more sensitively distinguished from wild type *TNFAIP3* function by analyzing NF-κB reporter gene activity in response to TLR or BCR signaling (18, 52). However, some missense variants did not show significant difference from wild type *TNFAIP3* using the methods mentioned above (18). One must carefully interpret whether such variants represent low-penetrance mutations, polymorphisms that contribute mildly to the development of HA20, or polymorphisms that contribute to other diseases. *TNFAIP3* Phe127Cys (rs2230926) is a single-nucleotide polymorphism associated with development of autoimmune diseases such as rheumatoid arthritis, SLE, and type 1 diabetes mellitus (DM) (53, 54). However, this variant showed no significant disruption of the inhibitory effects of A20, and it has not been evaluated as a pathogenic mutation for onset of HA20 in ClinVar (https://www.ncbi.nlm.nih.gov/clinvar/). It cannot be concluded that *TNFAIP3* variants identified by comprehensive genetic analysis are pathogenic based solely on allele frequencies and patient symptoms; validation by functional analysis is required. Furthermore, more sensitive and specific methods for functional analysis of *TNFAIP3* variants of unknown significance are required for future studies.

There is no standard treatment protocol for HA20. Various treatments are used in both East Asia and other regions, including colchicine, systemic corticosteroids, disease-modifying drugs, and molecular targeted therapies. The clinical severity of HA20 varies from mild to severe; responses to treatment also vary significantly. It has been reported that colchicine is effective to some extent in mild cases of HA20 (3, 10, 30, 37). In severe cases with poor response to colchicine, biological drugs are administered such as anti-TNF-α agents, anti-IL-1 agents, and anti-IL-6 agents. The rationale for such interventions is based on the increased production of proinflammatory cytokines such as TNF-α, IL-1β, IL-18, and IFN-γ-induced protein 10 in sera and stimulated peripheral blood mononuclear cells from patients with HA20 (3, 10). These therapies are effective in suppressing systemic inflammation in patients with other diseases. Anti-IL-1 agents were administered to one patient with HA20 in East Asia and were not effective; however, these agents were reported to be effective in some patients in other regions (3, 30, 35, 39, 40). Anti-IL-6 agents have been used less frequently to treat HA20 than anti-TNF-α and anti-IL-1 agents, but have been reported to be effective in a subset of patients (32). The efficacy of anti-IL-6 agents may relate to elevation of serum IL-6 during the active phase of disease (10). The possibility of the secondary ineffectiveness of biological drugs due to production of antibodies for them, especially anti-TNF-α agents, should be considered because severe HA20 patients complicated with autoimmune diseases are expected to have both autoinflammatory conditions and increased antibody production. Among molecular targeted therapies, JAK inhibitors were reported to be effective in patients with HA20 whose ISG expression was elevated in the active disease phase (38). Thus, administration of JAK inhibitors may become a more prominent treatment strategy. Although it has been conducted in only two patients with HA20 to date, HCT was effective in patients resistant to various treatments including several biological agents and led to remission of their inflammatory symptoms (31, 47). By contrast, a patient who received autologous HCT because of central nervous system vasculitis relapsed after 18 months of remission and various immunosuppressive agents were reinitiated (30). The first case of allogeneic HCT was a 14-year-old English boy. He had complex clinical features including development of insulin-dependent DM, cell depletion, hepatitis, enteropathy, and interstitial lung disease. Because his symptoms were refractory to treatment with prednisolone, sirolimus, tacrolimus, infliximab, and rituximab, he received HCT. Following HCT, he entered complete remission from all autoimmune disorders except DM. The second case was a 6-year-old Japanese boy. He had frequent fever, arthritis, psoriasis, aortic regurgitation, bowel disease, and genital ulcers. His symptoms were refractory to treatment with immunosuppressive drugs including prednisolone, cyclosporine, tocilizumab, anti-TNF-α agents, canakinumab, and tofacitinib. He underwent HCT and his autoinflammatory symptoms improved. However, some of his symptoms, such as severe aortic regurgitation and adrenal insufficiency, persisted because of long term administration of steroids. HCT might be an effective strategy for patients with treatment resistant HA20. However, it should be note that not all symptoms improve following HCT and that pre-existing organ damage will remain, potentially leading to symptom relapse. Because HA20 presents with various symptoms and severity, information on more cases is needed to establish appropriate treatment strategies.

## Conclusion

We summarized the symptoms and treatments of patients with HA20 in East Asia and other regions. Patients in East Asia had significantly higher rates of recurrent fever and lower rates of typical BD symptoms including genital ulcers and skin rashes. Patients in East Asia were less likely to have autoimmune disease complications. Differences between patients with HA20 in East Asia and other regions may relate to variation in the frequencies of other modifier genes and/or environmental factors. Future studies will need to establish functional analysis methods to validate the pathogenicity of *TNFAIP3* variants to enable rapid diagnosis of HA20 and development of appropriate treatment strategies.

## Author Contributions

All authors listed have made a substantial, direct and intellectual contribution to the work, and approved it for publication.

## Funding

This study was supported by MEXT KAKENHI (grant number JP21K07770), Health and Labour Science Research Grants for Research on Intractable Diseases from the Ministry of Health, Labour and Welfare of Japan (grant numbers 20316700 and 20317089), and AMED (grant number JP20ek0109480).

## Conflict of Interest

The authors declare that the research was conducted in the absence of any commercial or financial relationships that could be construed as a potential conflict of interest.

The handling editor declared a past collaboration with the authors TK, SK, and HO.

## Publisher’s Note

All claims expressed in this article are solely those of the authors and do not necessarily represent those of their affiliated organizations, or those of the publisher, the editors and the reviewers. Any product that may be evaluated in this article, or claim that may be made by its manufacturer, is not guaranteed or endorsed by the publisher.

## Abbreviations

BD, Behçet’s disease; BCR, B-cell receptor; DM, diabetes mellitus; EBV, Epstein–Barr virus; HA20, A20 haploinsufficiency; HCT, hematopoietic cell transplantation; HLA, Human Leukocyte Antigen; IKK, IκB kinase; IL, interleukin; IRF, interferon regulatory factor; ISGFBD, International Study Group for Behçet’s disease; ISGs, IFN-inducible-genes; JAK, Janus kinase; NF, nuclear factor; OTU, ovarian tumor; SLE, systemic lupus erythematosus; TBK1, TANK-binding kinase 1; TLR, Toll-like receptors; TNF, tumor necrosis factor; TRAF, TNF receptor associated factor; ZnF, zinc finger.
